# What opportunities exist for the governance of private sector engagement in mixed health systems in the African Region?

**DOI:** 10.7189/jogh.15.03038

**Published:** 2025-08-22

**Authors:** Michelle Amri, Omar Sam, Muriel Anye, Zandile Zibwowa, Humphrey Cyprian Karamagi, Juliet Nabyonga-Orem

**Affiliations:** 1 The W. Maurice Young Centre for Applied Ethics, School of Population and Public Health, University of British Columbia, Vancouver, British Columbia, Canada; 2 Office of the Assistant Regional Director, World Health Organization Regional Office for Africa, Cité du Djoué, Brazzaville, Republic of Congo; 3 Namibia Country Office, World Health Organization, Windhoek, Namibia; 4 Centre for Health Professions Education, Faculty of Health Sciences, North-West University, Potchefstroom, South Africa

## Abstract

Given the significant role of private actors in health across the African Region, there is a need to interrogate how private sector engagement (PSE) is governed. We identify three key opportunities for strengthening the governance of PSE in mixed health systems in the African Region. This work draws on a programme of activities conducted by the World Health Organization Regional Office for Africa, including a multi-country stakeholder survey, a regional meeting with participants from the public and private sectors, and a descriptive case study of the governance environment. We draw on a governance framework focused on three governance inputs – participation, consensus orientation, and strategic vision and policy design – to present opportunities. First, participation can be strengthened by formalising mechanisms for dialogue and consultation, creating partnerships, and defining roles and responsibilities. Second, better consensus orientation can be sought through aligning sectoral goals, such as through deploying champions and improving understandings of health equity. Third, strategic vision and policy design can be advanced by better integrating the private sector into mixed health systems. Integration can improve both data sharing in national health information systems and oversight. Moving forward, addressing how health equity can be re-centred and improving collaboration across sectors are essential.

Improving private sector engagement (PSE) in the governance of mixed health systems has been part of the programme of work of the World Health Organization (WHO) in the Africa Region over the past few years. We understand PSE in health governance to mean that governments focus on governing the whole system – that is, both the public and private sectors [1]. Mixed health systems are often characterised by out-of-pocket payments due to low insurance coverage and the dominance of private provision within an environment encompassing both privately and publicly financed government health service delivery [2]. Although private services are not typically more efficient, accountable, or medically effective than public services in low- and middle-income countries [3], healthcare in such contexts is delivered by mixed health systems [2], largely because publicly-funded health systems are generally chronically underfunded. Therefore, it is important for the public sector to engage the private sector for service delivery through meaningful PSE in mixed health systems [1]. It is thus crucial for the WHO to be pragmatic in seeking health for all [4]. Strengthening the governance of PSE in mixed health systems against the backdrop of broader efforts to improve Universal Health Coverage is therefore being pursued.

We understand governance as encompassing ‘formal institutions, accountability, trust, and legitimacy’ [5]. In other words, our interest is in governance inputs, processes, and outcomes [6]. To improve outcomes (*i.e.* responsiveness, equity, and efficiency) and governance processes (*i.e.* delivery of health services), we can interrogate governance inputs, which concern ‘how and by whom are the institutions and rules governing he health system constructed’ [7]. These governance inputs are participation, consensus orientation, and strategic vision and policy design [7]. Although we recognise that these outlined aspects can be understood as processes (*i.e.* how things are done), we refer to them as inputs to align with the framework we are referring to [7].

‘Good’ governance, recognising that there is no one definition of what this entails [8,9], ‘is synonymous with sound development management’ per the World Bank [10]. Here we understand ‘good’ governance is a normative concept, whereby certain aspects are deemed ‘bad’ (*e.g.* corruption), while recognising that there is no singular way to achieve good governance given that contextually appropriate practices should be employed. Further, some principles of good governance include accountability [11,12], transparency [12,13], effectiveness [11,13], coherence [11], strategic vision [13], equity [12,13], and notably, participation [11–13], which is thought to be necessary for many other principles [14]. Striving for ‘good’ governance through these principles thus requires the participation of the private sector, or PSE, given the role the private sector plays in mixed health systems. Further, having strong stakeholder voice in governance, which includes the private sector, can lead to enhanced health system performance [15].

The PSE work we have undertaken – including conducting a survey [16], hosting a multi-country regional meeting, and producing a descriptive case study of the governance environment [17] – has illuminated the need for the public sector to carefully consider the status of PSE and decide on how it wants to proceed. In detail, the online survey was developed based on the WHO’s six governance behaviours and distributed to 273 key individuals from ministries of health and their partner organisations, private sector institutions and initiatives in countries, and development organisations [16]. There were 81 respondents from across 13 countries (Angola, Burkina Faso, Burundi, Cabo Verde, Comoros, Congo, Côte d’Ivoire, Kenya, Mauritania, Nigeria, Senegal, Sierra Leone, and South Sudan), reflecting a response rate of 30% [16]. The descriptive case study was based on both a survey of stakeholders and presentations and deliberations at a WHO-hosted multi-country consultation in the African Region on PSE to advance progress towards Universal Health Coverage [17].

We have observed a disconnect between the public sector’s consideration of the private sector and, the private sector’s consideration of the public sector in the African Region. For instance, when assessing national health policies or health sector strategies of countries whose stakeholders were both invited to complete the survey and attend the meeting, the private sector is arguably included in the national health policy with some specificity on entities and roles in 12 country plans [16]. There was also strong awareness among key stakeholders about this inclusion of the private sector in national plans (88%) [16]. Yet despite this public sector awareness and inclusion of the private sector in policies and strategies, 51% of the stakeholders indicated that the national health policy is not used or is used in a limited way to guide the private sector, while 45% felt that the private sector does not engage the public sector [16]. Overall, how PSE is sought and accomplished must be afforded greater consideration, with particular attention paid to long-term ramifications. For this reason, we observe three key opportunities for countries around PSE in health governance in the African Region based on our work on PSE in the African Region and aligned with the governance inputs specified above. Each of these opportunities are discussed in depth below.

## PARTICIPATION: FORMALISE MECHANISMS FOR DIALOGUE AND CONSULTATION, CREATE PARTNERSHIPS, AND DEFINE ROLES AND RESPONSIBILITIES

First, starting with the governance input of participation, there is a need to establish formal structures that facilitate regular engagement between the public and private sectors to improve participation. Hosting periodic meetings for joint planning is one example of how dialogue can be fostered. Another is to establish public-private coordination platforms, particularly given that 82% of stakeholders indicated that such platforms did not exist, are available but not used, or used on an ad hoc basis [16]. In fact, 83% of participants at the meeting indicated that hosting a PSE forum would be of interest to them. However, in the development of such platforms, attention should be paid to dimensions of political economy, such as structural barriers, power asymmetries, and competing incentives.

There is also a need to create partnerships and define roles and responsibilities. In the case of Mauritania, the COVID-19 pandemic demonstrated that the two sectors need to work together, but the country is lacking a proper framework that contains well-defined roles for collaboration between the two sectors – especially in the case of the private sector using public personnel. Thus, it is important to define the roles and responsibilities of each sector, such as through memoranda of understanding and partnership agreements to enhance synergies. The agreements should outline the obligations of each party and be disseminated to the private sector. The memoranda of understanding between the Kebbi State Government in Nigeria and the United States Agency for International Development, which was performance-based, can be looked to as an example to guide the development of such agreements [18]. It featured quarterly and biannual reviews that tracked indicators through a dashboard, ultimately leading to improved monitoring, health sector work plan harmonisation, and data quality [18].

Because most meeting participants indicated that no PSE agreements are being signed with health districts to improve collaboration at the operational level, as is being done in Senegal, there is an opportunity to establish such partnerships. Partnerships can also promote the sharing of resources, capacities, and skills, given that this is an opportunity identified by 26% of stakeholders who felt that there is no such sharing [16]. It can also be established for the provision of services to essential and underserviced areas.

## CONSENSUS ORIENTATION: ALIGN SECTORAL GOALS

Second, in investigating consensus orientation, focussed on involving stakeholders for goal-setting and policy design [7], there is a need to better align sectoral goals. This could be tackled through managing competing and conflicting sectoral interests. Notably, 21% of stakeholders felt that population or civic interests are not mentioned as a part of PSE, and only 10% of them felt that population or civic interests are specified as a part of PSE [16]. In fact, only 8% of stakeholders felt that there are measures in place that are used to mitigate competing and conflicting sectoral interests [16]. It is also worth noting that the meeting participants observed how some not-for-profit organisations behave like for-profit companies. Thus, incentive structures for private sector provision in certain domains, such as rural areas with greater unmet need, can be explored.

Consensus orientation through aligning sectoral goals can also be improved through negotiations, champions, and deeper understandings of health equity. First, champions can be drawn on to manage these differing interests, given that 48% of stakeholders indicated that none are currently used in their respective settings [16]. Champions in health have gained broad acceptance over the past few decades and have been found to be important positive influences on implementation effectiveness [19]. Second, improving understanding of health equity may serve as one point of investigation and action. Because understandings and approaches to health equity and health inequities vary, how we understand these terms are presented in **Table 1**. Despite 81% of stakeholders indicating that health equity is always a priority in their own work, 19% of them indicated that it is either not a priority, sometimes a priority, or were unsure [16]. Deeper understandings of what health equity entails and how it can be actioned may help improve this lack of prioritisation.

**Table 1:** Select definitions of health equity and health inequities

Term	Definition
Health equity [20]	*Health equity means that everyone has a fair and just opportunity to be as healthy as possible. This requires removing obstacles to health such as poverty, discrimination, powerlessness, and their consequences – including lack of access to good jobs with fair pay, safe environments, and quality education, housing, and healthcare. […] For the purposes of measurement, health equity means reducing and ultimately eliminating disparities in health and its determinants that adversely affect excluded or marginalized groups.*
Health inequities [21]	*The term ‘inequity’ has a moral and ethical dimension. It refers to differences which are unnecessary and avoidable, but in addition are considered unfair and unjust.*

## STRATEGIC VISION AND POLICY DESIGN: BETTER INTEGRATE THE PRIVATE SECTOR INTO MIXED HEALTH SYSTEMS

Third, in the pursuit of strategic vision and policy design, which is focussed on ensuring institutions are conducive to the attainment of health policy goals [7], there is a need to better integrate the private sector into policy design. This does not entail the private sector directing public sector efforts, but rather that the latter considers the role of the former in achieving its goals. In fact, 20% of stakeholders indicated that there is no evidence of private sector integration in the health system, and only 7% reported that there is a defined national health policy or strategy monitoring mechanism in place that includes the private sector that is consistently used [16]. This is despite 95% of stakeholders noting that the private sector either partially or fully delivers services covered in essential health packages (among those whose countries have essential health packages) [16]. Coupled with 77% of stakeholders noting that there are either no guidelines available or that guidelines are available, but inconsistently used to align public and private providers, there is an evident need for better integration [16].

Integration can be improved for both data sharing in national health information systems and in terms of oversight. First, only 17% of key stakeholders felt that there was either moderate or universal reporting by private sector entities in national health information systems [16]. Thus, there is a need to synchronise data collection and use between government and private stakeholders. Second, integrating private sector efforts through oversight and monitoring mechanisms can also be improved. In fact, only 19% of stakeholders felt that there was a comprehensive regulatory framework in place for the private sector [16]. However, limited private sector compliance with regulations and rules can be due to limited or no public sector capacity, as was felt by 54% of stakeholders [16].

## CONCLUSIONS

We believe these three opportunities reflect the importance of ‘good’ governance and appropriate governance inputs of participation, consensus orientation, and strategic vision and policy design. These inputs align with governance prerequisites of institutions, management capacities, and culture to collaborate, which are noted as being necessary to allow effective partnerships and delivery designs that target those who are worse off [22]. Although tensions exist between public policy goals and private sector incentives and motivations, good governance can support the alignment of actors. Governments must reconsider the role of the private sector in the health system, in relation to new stakeholders that emerge through new policy processes, and in countries with decentralised health systems. Therefore, to steward the whole health sector, national health authorities need to have a comprehensive view on healthcare provision, including various governance challenges, such as political instability, corruption, and insufficient infrastructure.

Because PSE in health is not unique to the African Region, experiences from other regions can be drawn on to inform actions taken, such as experiences from the Eastern Mediterranean Region. In the Eastern Mediterranean Region, for-profit private providers offer 53% of inpatient services and 66% of outpatient services [23]. In 2015, 11.8% of the population in the Eastern Mediterranean Region faced financial hardship – defined as spending more than 10% of resources as direct out-of-pocket payments – which increased to 12.5% in 2017 [24]. Yet despite the contribution of the private health sector to service delivery, it is seldom included in national health systems planning and is not sufficiently regulated, which results in high out-of-pocket payments and low service coverage. The Eastern Mediterranean Region is the only WHO region that has an endorsed regional resolution for effective PSE in health, yet the operationalisation has been hindered by COVID-19. The WHO Eastern Mediterranean Regional Office is now seeking to engage the private health sector through a four-pillar approach that seeks to assess, govern, partner, and learn. There is an opportunity to relay these future experiences around best practices and challenges to stakeholders to promote knowledge translation and exchange, recognising unique contexts at play and no one-size-fits-all approach.

Moving forward, one key challenge envisioned for PSE in the African Region is around how health equity can be sought. Not only is health equity not prioritised by all, but there are different understandings of what health equity entails, which can further complicate aligned action [25]. A study of high-level African policymakers demonstrates this well, highlighting that policymakers’ views aligned with certain theories and that these theoretical underpinnings were tied to themes [26]. For example, ‘a utilitarian-minded policymaker may be focused on a widespread vaccination campaign, whereas a Rawlsian-aligned policymaker may focus on a targeted approach to reach communities that have lower vaccination rates, and a Senian-aligned policymaker may focus on health literacy programmes targeted at addressing vaccine-hesitant individuals within communities with lower vaccination rates’ [26]. Given domains of challenges expressed by high-level policymakers for better incorporating equity around: understanding health equity, governance, resources, and lack of data [27], similar challenges may persist for those working within the private sector that will need to be addressed across both sectors. Notably, because 19% of stakeholders indicated that health equity is either not a priority, sometimes a priority, or were unsure [16], this raises points of consideration both around how public-private efforts can re-center health equity in joint multisectoral efforts [28,29] – given that equity can even be sidelined in initiatives that are focussed on improving health equity [30] – and how competing views and understandings of health equity can be managed [25,31–37].
